# Positive soil responses to different vegetation restoration measures in desert photovoltaic power stations

**DOI:** 10.3389/fpls.2025.1607404

**Published:** 2025-05-27

**Authors:** Ruibing Meng, Zhongju Meng, Ruiting Jia, Haonian Li, Jiale Cai, Yue Gao

**Affiliations:** ^1^ College of Desert Control and Engineering, Inner Mongolia Agricultural University, Hohhot, China; ^2^ Key Laboratory of Aeolian Physics and Desertification Control Engineering from Inner Mongolia Autonomous Region, Inner Mongolia Agricultural University, Hohhot, China; ^3^ Key Laboratory of Desert Ecosystem Conservation and Restoration, State Forestry and Grassland Administration of China, Inner Mongolia Agricultural University, Hohhot, China; ^4^ Inner Mongolia Academy of Forestry Sciences, Hohhot, China

**Keywords:** photovoltaic, vegetation restoration, soil nutrients, desert, soil quality index

## Abstract

Scientific and reasonable vegetation restoration plays a pivotal role in enhancing soil quality, boosting ecosystem services, and ensuring the long-term stable operation of photovoltaic (PV) power stations in desert regions. To elucidate the response mechanisms of soil under different vegetation restoration implemented in PV power stations located in sandy areas, this study selected the PV power plant in Duguitala Township of the Hobq Desert as a representative research site. A systematic evaluation was conducted on the effects of four artificial vegetation restoration strategies, namely, *Leymus chinensis* (LC), *Glycyrrhiza uralensis* (GU), *Artemisia ordosica* (AO), and *Hedysarum scoparium* (HS) under panels and between panels. This analysis aimed to clarify the influence of different vegetation restoration approaches on soil quality in sandy regions and their underlying mechanisms. The findings revealed that these vegetation restoration measures significantly impacted soil texture, bulk density (BD), soil porosity (SP), soil water content, and water retention capacity. Specifically, LC and GU markedly improved soil physical structure and water retention capacities. Vegetation restoration substantially enhanced soil nutrient accumulation, with LC achieving the highest levels of multiple soil nutrient indices (total nitrogen (TN), total phosphorus (TP), and available potassium (AK)), HS exhibiting the highest level of available phosphorus (AP), and GU demonstrating superiority in total potassium (TK). These diverse vegetation restoration strategies exhibited potential advantages in improving soil fertility and promoting nutrient cycling at locations under PV panels. The soil quality index (SQI) showed that the effectiveness of the different vegetation measures in enhancing soil quality was ranked GU>LC>HS>AO>CK. This study not only provides robust theoretical support for ecological restoration in desert PV plants, but also offers practical experience applicable to vegetation restoration efforts in similar ecological environments, thereby possessing significant ecological and practical value.

## 1 Introduction

The global transition toward low-carbon and renewable energy systems has positioned solar energy as a critical solution to address energy crises and environmental degradation, owing to its cleanliness, low noise, accessibility, and minimal maintenance requirements (Binz et al., 2017; Sinke, 2019; Pourasl et al., 2023). Particularly in desert regions with abundant light and heat resources, the construction of large-scale photovoltaic (PV) power stations not only facilitates renewable energy development but also offers new opportunities for ecological restoration (Chang et al., 2020; Chen et al., 2024). However, these extreme conditions (e.g., high temperature, drought) not only threaten PV station stability but also hinder ecosystem restoration (Tang et al., 2021b). Consequently, balancing the relationship between renewable energy development and ecological protection while devising scientifically sound ecological restoration strategies has emerged as an urgent priority for advancing the PV industry in desert environments.

The extensive deployment of PV arrays alters surface energy balances and hydrological cycles, with shading effects and wind modulation influencing local microclimates (e.g., light availability, air temperature, and soil moisture) (Ezzaeri et al., 2018; Javed et al., 2020; Tang et al., 2021a; Yue et al., 2021b; Li et al., 2023). These changes introduce additional complexities for ecological restoration. Importantly, the PV-induced microclimate modifications are not uniform across the site. Marked spatial heterogeneity exists between the areas under the panels (shaded) and those between panels (exposed), with significant differences in light, temperature, and moisture conditions (Sansaniwal et al., 2018). On one hand, the shading effect of PV panels may mitigate surface temperature fluctuations and reduce evaporative losses, thereby creating more favorable growth conditions for certain drought-tolerant plant species and increasing vegetation density in shaded areas (Yue et al., 2021b). On the other hand, altered light and temperature conditions could decrease photosynthetic efficiency, potentially reducing vegetation density and biomass accumulation. Conversely, exposed areas between panels receive higher levels of solar radiation and elevated temperatures, resulting in distinct patterns of biomass partitioning (Meng et al., 2025). The environmental heterogeneity between shaded and exposed zones not only influences plant growth but also profoundly implications the functionality and stability of desert ecosystems. While prior studies have examined the impact of large-scale PV station construction on microclimate conditions and sand-fixing vegetation growth patterns, they often assume uniform effects across the entire installation, neglecting potential spatial variations between different zones. Addressing these gaps is critical for refining ecological restoration strategies and optimizing vegetation management in desert PV systems. However, most existing studies have focused on the overall effects of PV construction without explicitly comparing the ecological responses under and between panels, thereby failing to capture the fine-scale spatial variation in microenvironments. This limits the development of microclimate-adapted restoration strategies.

Existing research on desert PV power stations has primarily focused on controlling wind-sand activity through measures such as vegetation restoration and biological soil crusts (El Chaar et al., 2011; Orr, 2016). Among these, vegetation restoration stands out as one of the most cost-effective, ecologically beneficial, and sustainable methods for desert ecosystem rehabilitation (Uldrijan et al., 2021; Cai et al., 2024). Consistent with this, some researchers have demonstrated that appropriate vegetation deployment within PV arrays can effectively reduce wind erosion, enhance soil moisture retention, and promote organic matter accumulation, thereby improving overall ecological conditions (Choi et al., 2020; Javed et al., 2020). The above-ground portion of the vegetation increased surface roughness to reduce wind speed and wind erosion, while the below-ground root system improved soil stability and contributed to nutrient accumulation and soil quality enhancement (Huang et al., 2018; Tang et al., 2021a; Wang et al., 2021). Additionally, plant residue decomposition contributes to carbon and nitrogen cycling, sustaining long-term soil fertility (Luo et al., 2024).

The dynamic interaction between vegetation and soil systems forms the foundation of ecological restoration, determining the stability and sustainability of degraded ecosystems (Randle-Boggis et al., 2020). Under the policy framework of “PV + Ecology,” governments at various levels have actively promoted vegetation restoration in desert PV areas (Ren et al., 2022). Through optimized spatial planning, agro-photovoltaic integrated systems achieve synergistic benefits by combining PV power generation with vegetation cultivation. The shading and humidifying effects of PV panels create favorable microenvironments for understory plant growth, enhancing both land productivity and ecological benefits (Liu et al., 2020; Knapp and Sturchio, 2024). Nevertheless, research indicates that the effects of different vegetation types on soil improvement vary significantly, depending on the vegetation type, growth characteristics and environmental adaptations (Kavga et al., 2018). For instance, leguminous plants enhance soil nitrogen content through biological nitrogen fixation, whereas graminoids excel in wind erosion control and soil stabilization due to their extensive root systems (Kong et al., 2010; Monnens et al., 2023). Additionally, certain xerophytic species (e.g., *Astragalus adsurgens*, *Glycyrrhiza uralensis*, etc.) exhibit strong ecological adaptability through root morphology plasticity, demonstrating high potential for desert PV restoration (Meng et al., 2025). Despite these advancements, current studies predominantly focus on single-species planting, lacking systematic evaluation of. multi-species combinations’ ecological restoration effects. Additionally, the integrated impacts of PV-induced microclimate changes on vegetation-soil interactions remain insufficiently understood. Bridging these gaps necessitates comprehensive research to quantify the impacts of diverse restoration measures on soil quality, elucidate vegetation-soil feedback mechanisms, and optimize ecological restoration strategies for desert PV environments.

To address these gaps, our study was conducted at a large-scale PV power station in Hangjin Banner, Ordos City, Inner Mongolia. Using bare sandy land as the control (CK), we investigated the soil improvement mechanisms under four artificial vegetation restoration measures: *Leymus chinensis* (LC), *Glycyrrhiza uralensis* (GU), *Artemisia ordosica* (AO), *and Hedysarum scoparium* (HS), both under panels and between panels. The specific objectives were to (1) quantify soil moisture dynamics under different restoration measures, (2) compare their effects on soil quality and structural stability, and (3) identify key soil quality indicators and their driving mechanisms in PV-assisted desert restoration. By integrating soil physicochemical properties and a Soil Quality Index (SQI), this study aims to determine the optimal vegetation restoration strategy for enhancing ecological recovery, soil quality, and ecosystem stability in desert PV systems. The findings will provide empirical support for vegetation management in operational PV plants, inform secondary sand hazard mitigation, and contribute to the sustainable coexistence of PV infrastructure and desert ecosystems.

## 2 Materials and methods

### 2.1 Study area description

**Figure 1:**
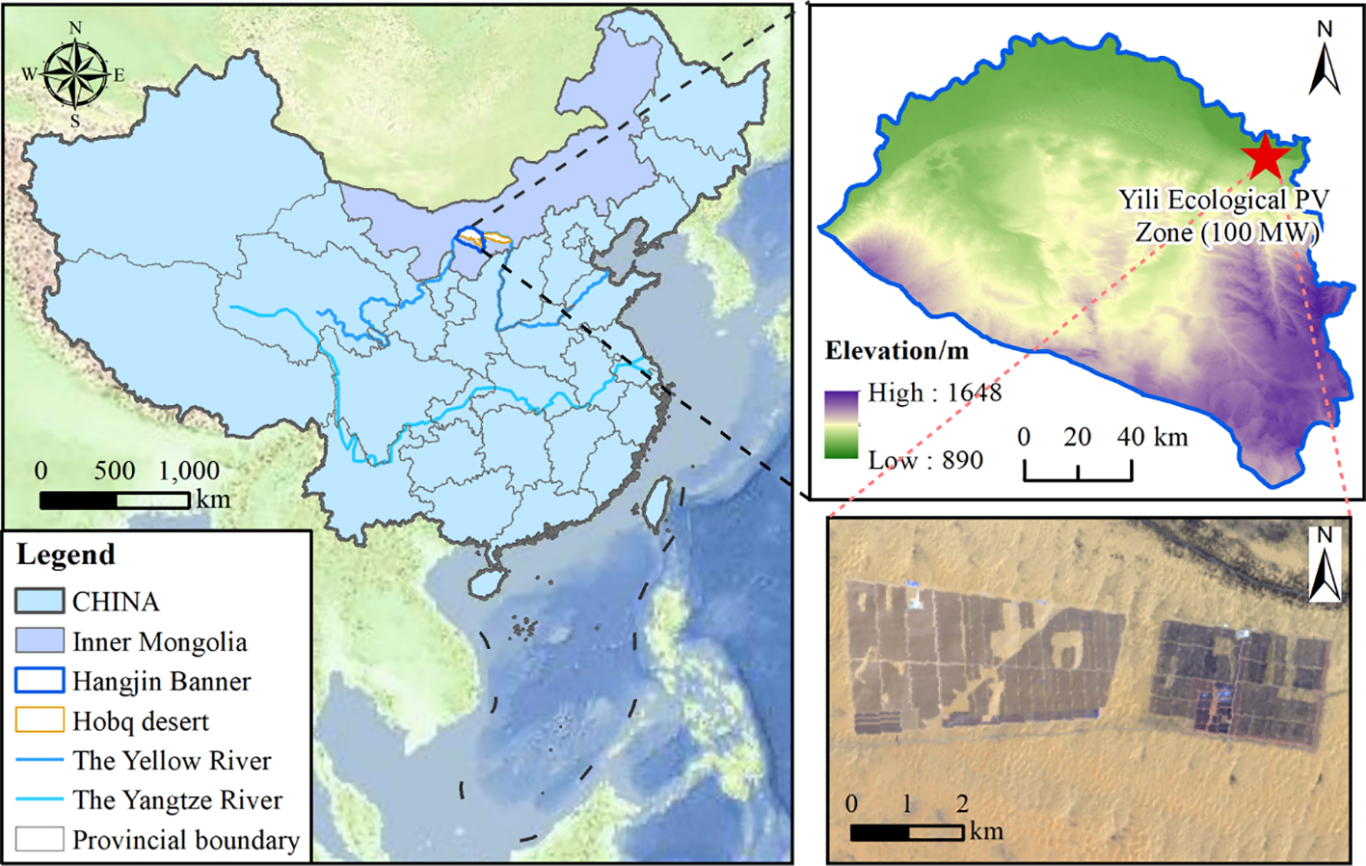
The location of the study area.

The study area is located in a PV power station situated on the northern edge of the Hobq Desert, within the Duguitala Industrial Park in Hangjin Banner, Ordos City, Inner Mongolia (37°20′—39°50′ N, 107°10′—111°45′ E), covering a total area of 6.67 km² (**Figure 1**). The region is characterized by a typical temperate continental monsoon climate with an elevation of 1136 m above sea level. The mean annual temperature ranges from 5 to 8°C, and the mean annual total solar radiation is 597.9 kJ·cm^-2^. The mean annual precipitation is between 150 and 400 mm, predominantly occurring from late June to early September, while the annual evaporation varies from 2100 to 2700 mm. Aeolian activity is concentrated between March and May, with a maximum instantaneous wind speed of 24 m·s^-1^ and an annual frequency of strong wind events ranging from 25 to 35 days. The prevailing northwesterly winds have shaped diverse dune landforms, including barchan dunes, barchan dune chains, and grid dune chains, with an overall low vegetation coverage, of which 60% of the sands are mobile dunes.

The study was conducted in the Hobq Desert (Duguitala Township), at the Yili Ecological PV Zone (100 MW), which commenced operation in 2016. Before the construction of the PV power station, the underlying surface primarily consisted of shifting sand dunes with a vegetation cover of less than 3%. The station comprises monocrystalline silicon photovoltaic panels arranged at an optimal tilt angle of 36°, facing south and oriented in an east-west direction. The spacing between adjacent PV panel arrays is 900 cm, with the upper and lower panel edges positioned at 270 cm and 35 cm above the ground, respectively. Each panel unit consists of two rows of 18 columns, with individual photovoltaic panels measuring 99 cm×195 cm, forming an overall module size of 400 cm×1800 cm. The total operational area of the PV station is 5.37 km². The entire PV station is uniformly covered with a red clay substrate.

### 2.2 Experimental design and sample collection

**Figure 2:**
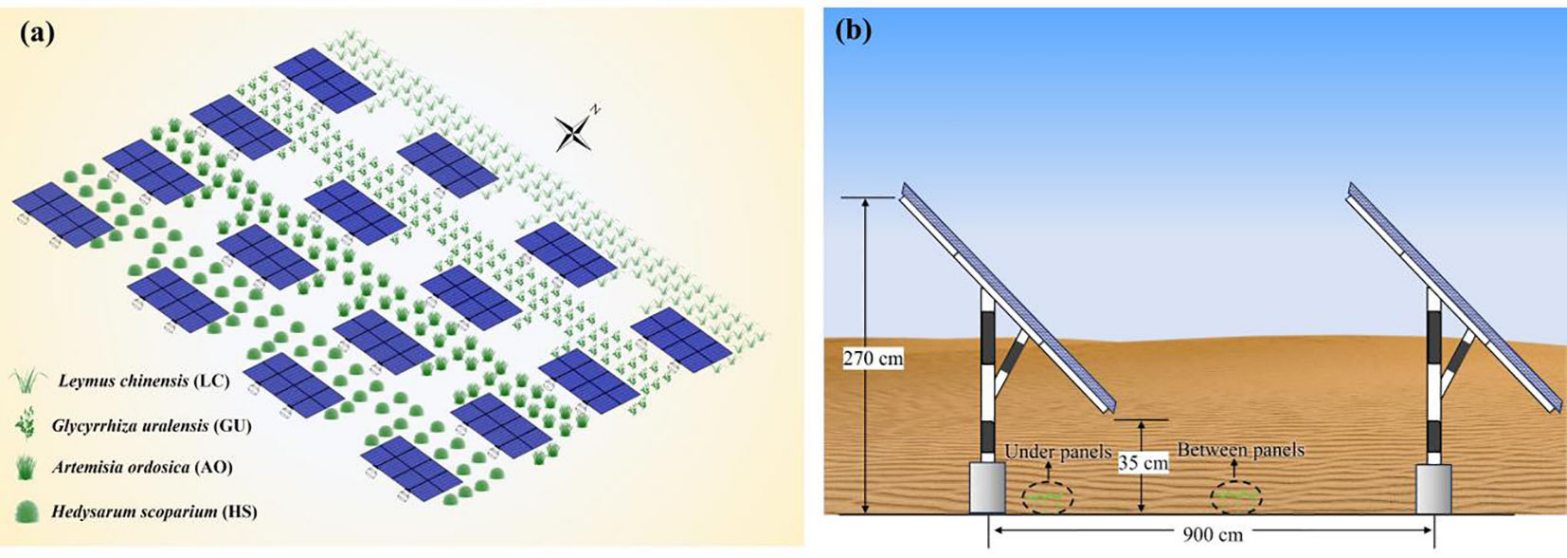
Experimental design of vegetation restoration sample plots for photovoltaic power station. **(a)** Distribution of different vegetation restoration sample plots **(b)** Soil sampling at different locations of desert photovoltaic panels.

To mitigate wind erosion and sand burial risks, four representative sand-binding plant species, *Leymus chinensis* (LC), *Glycyrrhiza uralensis* (GU), *Artemisia ordosica* (AO), and *Hedysarum scoparium* (HS), were selected in 2020 for vegetation restoration. These species were chosen based on their local dominance in arid and semi-arid steppe ecosystems, documented success in previous sand-fixation projects, and specific functional traits conducive to PV microclimate environments. Each experimental plot measured 33 m×54 m, corresponding to the layout of a standard PV panel unit in the studied station (**Figure 2**). This plot size ensures that the effects of vegetation restoration are evaluated under representative conditions of PV-induced microenvironments. The fundamental characteristics of the vegetation plots during the growing season are summarized in **Table 1**.

**Table 1:** Basic information on sample plot.

Restoration measures	Vegetation types	Planting time	Plant height/cm	Array spacing (m)
LC	*Leymus chinensis*	2020	63	0.3×0.4
GU	*Glycyrrhiza uralensis*	2020	11	0.3×0.4
AO	*Artemisia ordosica*	2020	145	1×1
HS	*Hedysarum scoparium*	2020	131	1×2
Moving sand dunes (CK)	NA	NA	NA	NA

The field experiment was conducted during the growing season in July 2022. To ensure the reliability of the results, sampling was performed under stable meteorological conditions with no precipitation events recorded within one week before and after sampling. Within each vegetation restoration plot, three 1 m × 1 m quadrats were randomly established for herbaceous species, and three 5 m×5 m quadrats for shrub species. Vegetation characteristics, including species composition, coverage, height, and canopy width, were systematically recorded. In addition, soil profiles beneath representative plant were excavated, and surface soil samples were collected using the cut-ring method. These samples were analyzed for soil bulk density, moisture content, and porosity. Moreover, approximately 500 g of soil was collected using polyethylene bags, transported to the laboratory, air-dried, and sieved to remove debris for further analysis of soil texture and nutrient content. To enhance experimental reliability, three replicate plots with morphologically similar and physiologically consistent plants were selected under the same PV panel row. The control plot (CK) consisted of non-vegetated mobile sand dunes outside the PV station, used to evaluate how vegetation restoration improves soil quality.

### 2.3 Sample analysis and experimental methods

Soil samples were transported to the laboratory for immediate determination of soil moisture content (MC) using the oven-drying method (105°C for 48 h). Bulk density (BD), saturated hydraulic conductivity (Ks) Equation 1, capillary water content (CWC) Equation 2, field water capacity (FC) Equation 3, and soil porosity (SP) Equation 4 were measured using the cutting ring method with a stainless steel cylinder (100 cm^3^ volume). Specifically, a pre-weighed cutting ring was used to collect undisturbed soil cores. A filter paper was placed at the perforated end of the ring, which was then secured with a rubber band to maintain structural integrity during handling. The soil-filled ring was immersed in water for 12 h to achieve saturation, with the saturated mass recorded as m_1_. Subsequently, the ring was placed on dry sand for 2 h, and the mass was measured as m_2_. After an additional 48 h of drainage on the sand bed, the mass was recorded as m_3_. Finally, the soil core was oven-dried at 105°C to constant mass (m_4_). The parameters were calculated as follows:


(1) $$\text{Ks}(\%)=\frac{{\text{m}}_{1}-{\text{m}}_{4}}{{\text{m}}_{4}}$$

(2) $$\text{CWC}=\frac{{\text{m}}_{2}-{\text{m}}_{4}}{{\text{m}}_{4}}$$

(3) $$\text{FC}=\frac{{\text{m}}_{5}-{\text{m}}_{6}}{{\text{m}}_{6}}$$

(4) $$\text{SP}=\frac{\text{Ks}\times \text{BD}}{1.0\text{g}/{\text{cm}}^{3}}$$

Determination of soil particle size composition with the Mastersizer 3000 laser particle sizer. The collected soil samples were air-dried, with plant residues and gravel removed, and large aggregates disintegrated. The processed soil was ground and passed through 20-mesh (0.9 mm) and 100-mesh (0.15 mm) nylon sieves for further analysis. Soil organic carbon (SOC) was determined using the dichromate oxidation-heating method. Available phosphorus (AP) was extracted using 0.5 M NaHCO_3_ and quantified via colorimetry. Available potassium (AK) was extracted using 2 M HNO_3_ and measured by flame photometry, while alkali-hydrolyzable nitrogen (AN) was determined using the alkali diffusion method. Total potassium (TK) was analyzed using sodium hydroxide fusion followed by flame photometry, whereas total phosphorus (TP) was measured using sodium hydroxide fusion-molybdenum blue spectrophotometry. All analyses were conducted in triplicate to ensure accuracy and reproducibility.

### 2.4 Calculation of soil quality index

A single soil parameter is insufficient to comprehensively assess changes in soil quality. Therefore, the Soil Quality Index (SQI) was employed to integrate multiple soil parameters for a comparative evaluation of different vegetation restoration types. Considering the frequency and representativeness of soil indicators in previous studies and the experimental conditions, 14 measured soil indicators were selected as the TDS. The Kaiser-Meyer-Olkin (KMO) test and Bartlett’s sphericity test were conducted to assess the suitability of the dataset for PCA. The KMO value was 0.705 (>0.6), and Bartlett’s test showed a significance level of p < 0.001, indicating that the selected indicators were appropriate for PCA. PCA was performed to obtain eigenvalues, variance contribution rates, and factor loading matrices for the principal components. The coefficients for each principal component were calculated by dividing the factor loadings of each indicator by the square root of the corresponding principal component’s eigenvalue. The weight (W_i_) of each indicator was determined as the proportion of its communality variance to the total communality variance of all indicators.

The minimum data set (MDS) is an approach used to evaluate soil quality by selecting the most essential variables that best represent soil function (Raiesi and Pejman, 2021). This method reduces the workload associated with data measurement and analysis while preserving the critical information needed for quality assessment (Raiesi, 2017; Gan et al., 2024). MDS selection can be based on expert judgment or statistical methods. In this study, principal components with eigenvalues ≥1 were selected. Among these, indicators with factor loadings within the top 10% of each principal component were considered high-loading indicators. If a principal component contained only one high-loading indicator, it was directly included in the MDS. If multiple high-loading indicators were present, Pearson correlation analysis was performed to assess their relationships. If the indicators were uncorrelated, all were retained in the MDS; otherwise, only the indicator with the highest loading was selected.

To validate the effectiveness of the MDS, a comprehensive factor loading calculation was conducted for all indicators in the TDS. The results of the TDS and MDS were then compared to evaluate the ability of the MDS to retain key information while simplifying the dataset. The calculation formula is as follows Equation 5 (Vasu et al., 2016; Zhu et al., 2024):


(5) $$N_{ik}=\sqrt{\sum_{1}^{k}(U_{ik}^{2}\lambda_{k})}$$

where *N_ik_* is the cumulative factor loading of indicator i across all k principal components, *U_ik_* is the loading of indicator *i* on principal component *k*, and $\lambda_{k}$ is the eigenvalue of the *k*th principal component.

The Soil Quality Index (SQI) Equation 6 was then calculated by a weighted summation of the linear scores and weight coefficients of each indicator (Paul et al., 2020):


(6) $$\text{SQI}=\sum_{\text{i}=1}^{\text{n}}{\text{W}}_{\text{i}}{\text{N}}_{\text{i}}$$

where *N_i_* and *W_i_* represent the linear score and weight coefficient of the *i*th soil indicator, respectively, and *n* is the total number of soil indicators in the dataset.

### 2.5 Data analysis

Soil property data were collected from the four different vegetation restoration treatments. Statistical analyses were conducted using SPSS Ver. 19. One-way analysis of variance (ANOVA) was employed to compare soil properties among the five treatments. Fisher’s least significant difference (LSD) test was used for mean separation at significance levels of P < 0.05 and P < 0.01. Pearson correlation analysis was performed to evaluate relationships among selected soil parameters. Principal component analysis (PCA) was conducted for factor extraction, and data processing was performed using Microsoft Excel. Three replicate samples were separately analyzed to ensure independent verification of laboratory measurements and statistical analysis.

## 3 Results

### 3.1 Changes in soil physical properties under different vegetation restoration patterns

As shown in **Table 2**, different vegetation restoration measures significant influenced soil texture, porosity, and moisture characteristics. Compared with other treatments, LC exhibited the highest silt sand content and the lowest clay content, suggesting its effectiveness in improving soil particle structure. BD and SP are critical indicators of soil permeability, aeration, and water-holding capacity. All four vegetation restoration measures reduced BD, with GU showing the lowest value under panels at 1.47g·cm-3, which was significantly lower than CK (*P < 0.05*), while SP was highest in HS. Vegetation cover plays a crucial role in regulating soil moisture content, as evidenced by previous studies (De Almeida et al., 2018; Shen et al., 2020). Our results revealed that LC, AO, and HS exhibited significantly higher MC than CK, whereas GU showed slightly lower water retention capacity compared to the other vegetation measures. Furthermore, MC was significantly higher in under panels than between panels for each plant measure, consistent with previous findings that PV panels exert cooling and humidifying effects (Yue et al., 2021a). Ks and CWC are key parameters reflecting soil water transmission and retention. Both LC and GU displayed significantly higher values for these parameters compared to other vegetation types and CK. Notably, under panels, LC achieved a Ks rate of 15.49%, significantly exceeding CK (13.39%). Additionally, GU exhibited the highest FC, likely attributable to its root distribution and soil moisture utilization strategy. GU was known for developing deep taproots and widespread lateral roots, enhancing its capacity for soil water absorption and storage (Zheng et al., 2018). These root characteristics likely contribute to its superior field capacity under PV panel shading conditions. These findings collectively indicate that: 1) CK exhibited the highest soil compaction and poorest soil functionality; 2) Both LC and GU demonstrated superior performance in enhancing SP, reducing BD, and improving hydraulic conductivity and water retention; 3) For AO and HS, some soil parameters did not differ significantly from CK, suggesting limited improvement in soil physical properties.

**Table 2:** Changes in soil physical properties between and under panels with different vegetation restoration measures.

Parament	Location	*Leymus chinensis* (LC)	*Glycyrrhiza uralensis* (GU)	*Artemisia ordosica* (AO)	*Hedysarum scoparium* (HS)	Control (CK)	F value
Clay (%)	Between panels	97.22 ± 0.10c	97.7 ± 0.08b	99.36 ± 0.36a	99.29 ± 0.35a	99.56 ± 0.28a	51.59**
	Under panels	97.81 ± 0.20c	98.84 ± 0.07b	99.47 ± 0.07a	99.24 ± 0.11a	99.56 ± 0.28a	53.61**
Silt sand (%)	Between panels	2.78 ± 0.10a	2.30 ± 0.08b	0.64 ± 0.36c	0.71 ± 0.35c	0.44 ± 0.28c	51.59**
	Under panels	2.19 ± 0.20a	1.16 ± 0.07b	0.53 ± 0.07c	0.76 ± 0.11c	0.44 ± 0.28c	53.61**
Bulk density (BD) (g·cm-3)	Between panels	1.53 ± 0.02bc	1.52 ± 0.01c	1.57 ± 0.11bc	1.60 ± 0.02ab	1.68 ± 0.02a	5.59
	Under panels	1.52 ± 0.02c	1.47 ± 0.02bc	1.56 ± 0.06bc	1.59 ± 0.04b	1.68 ± 0.06a	8.98*
Soil porosity (SP) (%)	Between panels	23.29 ± 0.44a	22.73 ± 0.80ab	21.59 ± 1.03b	22.79 ± 0.21ab	22.49 ± 0.37ab	4.27
	Under panels	23.55 ± 0.11a	20.87 ± 0.53c	22.47 ± 0.50b	24.11 ± 0.73a	22.49 ± 0.37b	18.86**
Moisture content (MC) (%)	Between panels	1.77 ± 0.05a	1.42 ± 0.42b	1.89 ± 0.35a	1.74 ± 0.06a	1.04 ± 0.26b	13.80**
	Under panels	2.87 ± 0.10a	1.58 ± 0.04d	1.93 ± 0.34b	1.90 ± 0.13b	1.04 ± 0.26c	55.93**
Saturated Hydraulic Conductivity (Ks) (%)	Between panels	15.23 ± 0.12a	14.96 ± 0.62ab	13.72 ± 0.55cd	14.25 ± 0.28bc	13.39 ± 0.34d	10.40*
	Under panels	15.49 ± 0.14a	14.22 ± 0.19b	14.40 ± 0.55b	15.17 ± 0.16a	13.39 ± 0.34c	20.55**
Capillary Water Content (CWC) (%)	Between panels	13.88 ± 0.04a	13.59 ± 0.27a	11.77 ± 0.69b	12.44 ± 0.23b	7.54 ± 0.81c	77.06**
	Under panels	13.65 ± 0.08a	12.94 ± 0.17a	11.32 ± 1.63b	12.76 ± 0.31ab	7.54 ± 0.81c	25.94**
Field Water Content (FC) (%)	Between panels	5.16 ± 0.0b	9.91 ± 0.16a	8.92 ± 1.14a	8.53 ± 0.60a	4.56 ± 0.40b	27.93**
	Under panels	4.66 ± 0.10b	8.78 ± 1.09a	7.42 ± 0.51a	8.76 ± 0.34a	4.56 ± 0.40b	8.55*

Values in each column with the same letter are not significantly (*P > 0.05*, LSD) different among the vegetation restoration measures. **Significant at the 0.01 level and * Significant at the 0.05 level.

### 3.2 Changes in soil chemical properties under different vegetation restoration patterns

**Figure 3:**
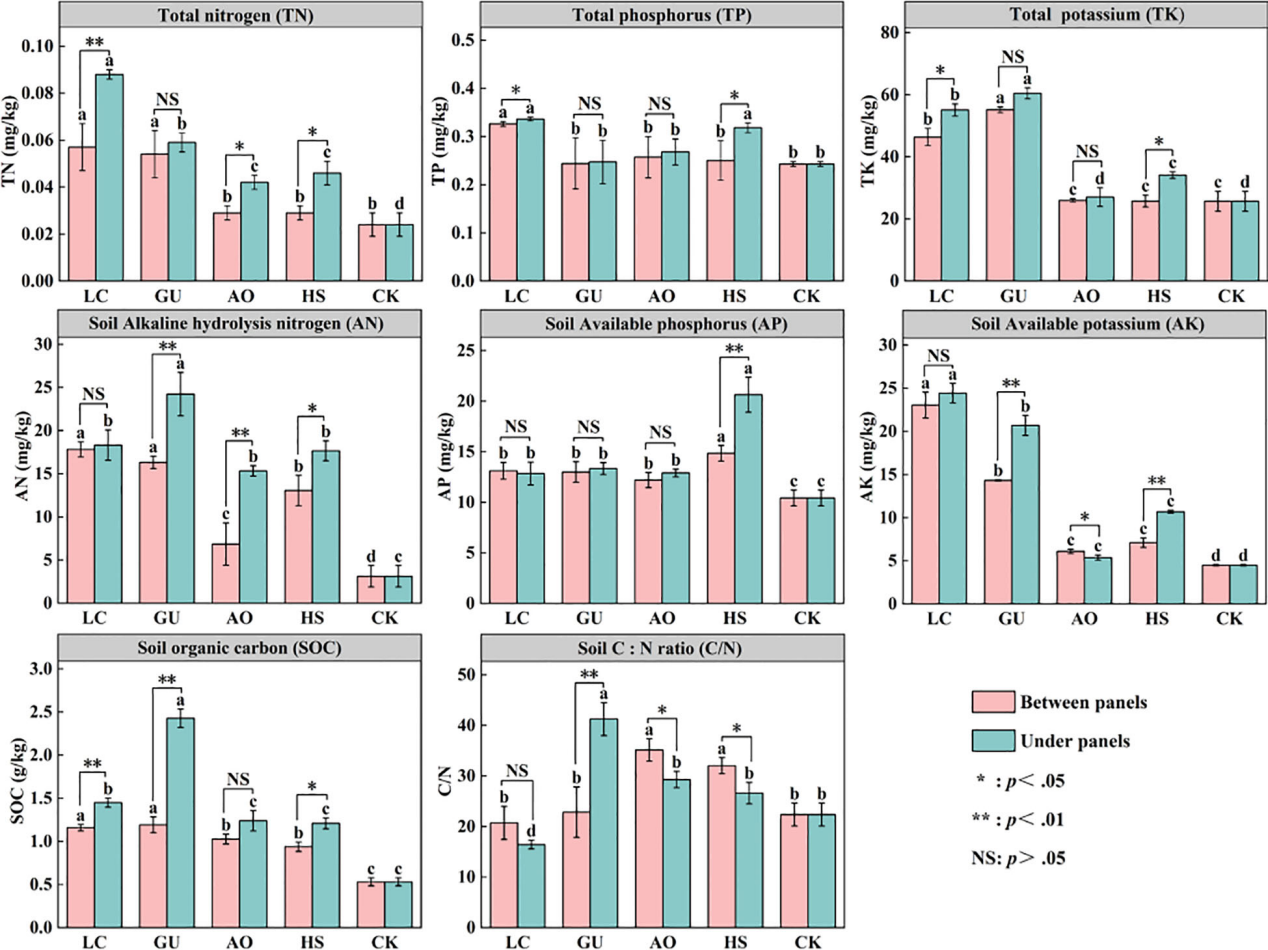
Distribution of soil nutrients under different vegetation measures. Values with the same letter were not significantly different between vegetation restoration measures (P > 0.05, LSD). NS indicates no significant difference in between panels and under panels for the same plant measure. **Significant at the 0.01 level, *Significant at the 0.05 level.

**Figure 3** illustrate that different vegetation restoration measures significantly altered key soil nutrient indicators, including TN, AN, AP, AK and SOC (*P<0.05*). Specifically, the LC treatment exhibited significantly higher TN, TP, and AK contents compared to other treatments (*p < 0.05*), indicating its strong potential for soil improvement. The HS treatment exhibited the highest AP content, while the GU treatment showed the greatest TK content. In contrast, the AO treatments demonstrated relatively lower soil nutrient accumulation, which may be related to species-specific differences in nutrient uptake, allocation, and cycling. In addition, all vegetation measures significantly increased SOC content. Compared to CK, the SOC content in LC and GU treatments increased by 146.23% and 241.51%, respectively. This further validated the synergistic mechanism of vegetation restoration to enhance soil organic carbon pools through apoptotic inputs and microbial-mediated carbon fixation (Shi et al., 2023). The C/N ratio analysis revealed that the GU treatment had the highest C/N ratio (41.24), suggesting potential nitrogen limitation, whereas the LC treatment exhibited a lower and more stable C/N ratio (16.43), indicating efficient nitrogen cycling. Notably, soil nutrient and SOC levels were generally higher under panels than between panels *(P < 0.05*), with the LC treatment showing the most pronounced effects. This correlates with the positive impacts of PV panel construction on the soil microenvironment beneath them, thereby enhances the soil amelioration effects of various vegetation restoration measures (Yue et al., 2021a).

### 3.3 Relationships between soil factors

**Figure 4:**
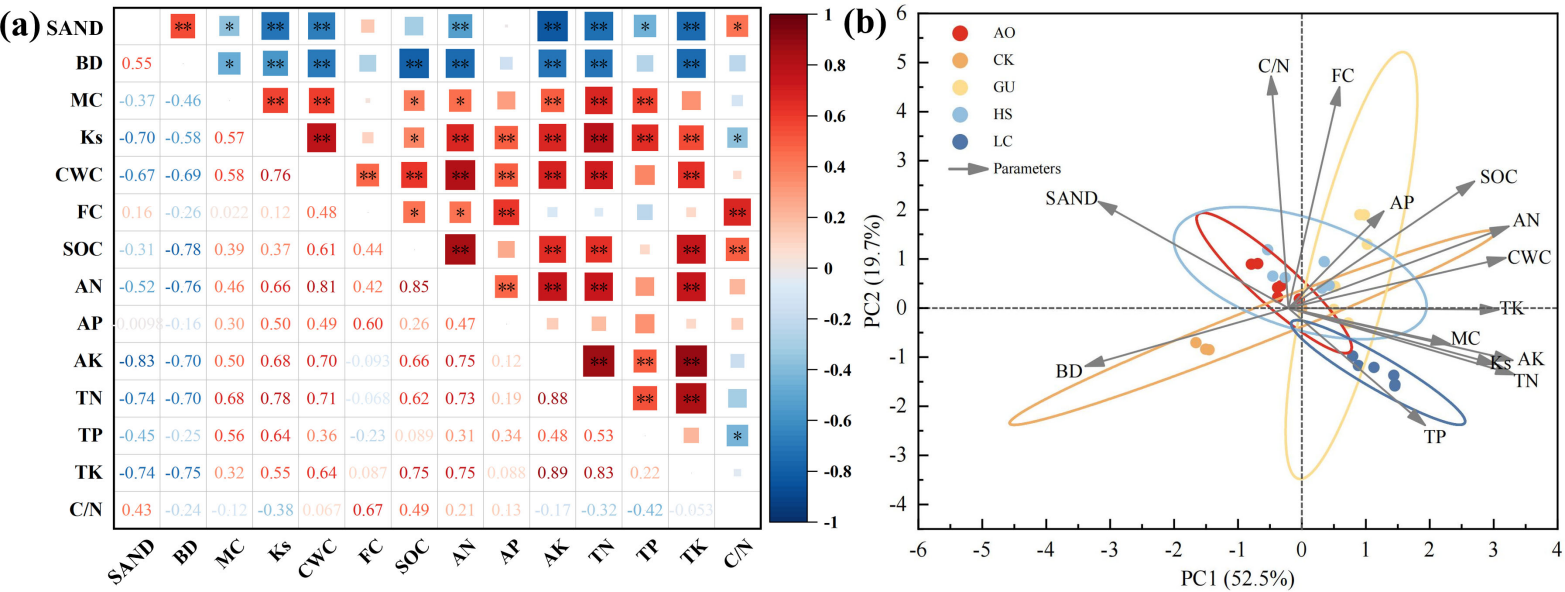
Comprehensive analysis of soil indicators for various vegetation restoration measures **(a)** Correlation analysis. **(b)** Principal component analysis. **Significant at the 0.01 level, *Significant at the 0.05 level.

The results of soil factor correlation analysis under each plant measure in PV power station at sandy area are shown in **Figure 4a**, which shows that the soil factors are related closely. Sand content exhibited significant positive correlations with BD and C/N ratio (*P<0.05*), but strong negative correlations with Ks, CWC, and soil nutrients (AN, AK, TN, TK) (*P<0.01*), indicating coarse-textured soils compromise water and nutrient retention. Both K_s_ and CWC showed positive associations with all nutrients (*P<0.05*), demonstrating improved hydraulic properties enhance nutrient mobility. Notably, SOC correlated positively with multiple soil nutrients and moisture indicators (*P<0.05*), confirming its pivotal role in coupling water retention and nutrient cycling. These findings collectively suggest that in desert PV ecosystems, mitigating sand dominance while increasing SOC can synergistically optimize soil structure, hydraulic function, and fertility.

### 3.4 Soil quality assessment

To evaluate soil quality under different vegetation restoration measures in a desert PV power station, we selected 14 key indicators reflecting soil properties: Sand, BD, MC, Ks, CWC, FC, SOC, AN, AP, AK, TN, TP, TK, and C/N ratio (**Figure 4b**). These variables constituted the TDS for soil quality assessment. PCA was employed to reduce data dimensionality, and the results showed that the first three principal components (PC1–PC3) accounted for a cumulative variance of 83.58% (**Table 3**), effectively capturing the primary variability in the dataset.

**Table 3:** Principal component analysis (PCA) of soil parameters.

Soil parameters	Symbol	Principal component			Weight of TDS	Weight of MDS
		PC1	PC2	PC3		
Sand content	Sand	-0.766	0.420	0.175	0.0678	0.1601
Soil bulk density	BD	-0.819	-0.229	0.271	0.0680	–
Soil moisture content	MC	0.650	-0.142	0.316	0.0464	–
Saturated hydraulic conductivity	Ks	0.836	-0.222	0.354	0.0746	0.1216
Capillary water content	CWC	0.875	0.199	0.178	0.0715	–
Field water content	FC	0.205	0.872	0.285	0.0756	0.1259
Soil organic carbon	SOC	0.749	0.500	-0.302	0.0771	–
Soil available nitrogen	AN	0.888	0.322	-0.020	0.0762	–
Soil available phosphorus	AP	0.384	0.382	0.749	0.0729	0.0858
Soil available potassium	AK	0.904	-0.206	-0.253	0.0790	0.1076
Soil total nitrogen	TN	0.910	-0.262	-0.083	0.0773	0.0892
Soil total phosphorus	TP	0.551	-0.462	0.454	0.0618	–
Soil total potassium	TK	0.847	-0.006	-0.427	0.0768	–
Soil C/N ratio	C/N	-0.070	0.815	-0.183	0.0748	–
Characteristic root	7.353		2.761	1.587	–	–
Variance contribution rates/%	52.525		19.718	11.334	–	–
Cumulative variance contributions/%	52.525		72.242	83.577	–	–

**Figure 5:**
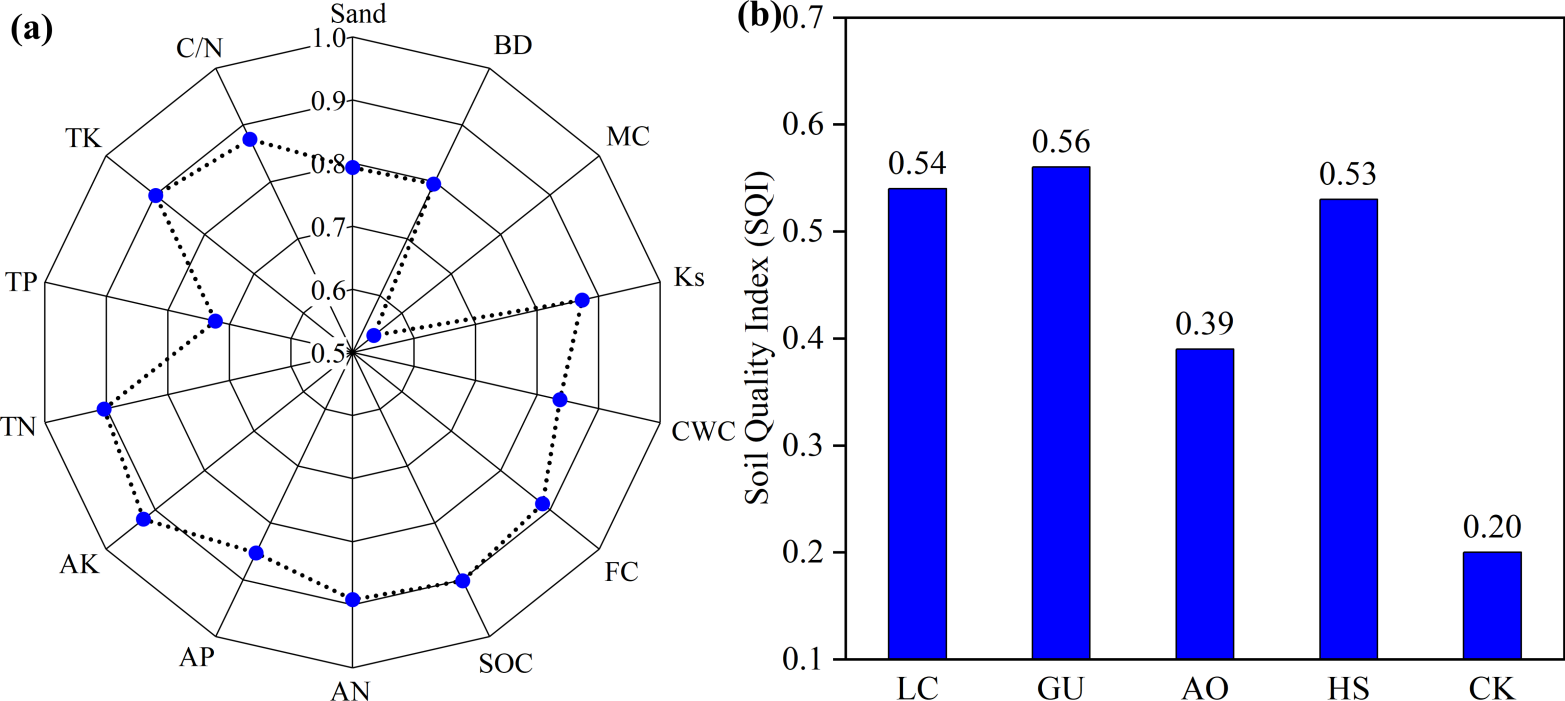
Assessment of soil quality index (SQI) under different vegetation restoration measures. **(a)** The weight of the soil parameters. **(b)** soil quality index.

Based on the PCA results, the MDS was constructed by selecting the indicators with higher factor loadings. When multiple high-loading indicators are present within a single principal component, we calculate their correlation coefficients to determine potential redundancy. If two high-loading variables exhibit a strong correlation, only the one with the highest loading is retained. If they are independent of each other, both are included in the MDS. In PC1, TN and AK exhibited the highest loadings (>0.9), highlighting their critical role in soil quality assessment. Additionally, Ks was included due to its strong association with water movement and vegetation-mediated moisture regulation. In PC2, both FC and C/N exhibited high loadings; however, since they are significantly correlated, only FC is retained. In PC3, AP has the highest loading. Besides PCA loadings, practical significance and conventional soil quality evaluation criteria were also considered. Although sand content did not exhibit the highest loading, it was incorporated into the MDS due to its significant influence on soil texture and water retention capacity. Ultimately, six core indicators were selected for the MDS: sand, Ks, FC, AN, AK, and TN. Since these indicators have different units of measurement, a membership function was applied to standardize the MDS, followed by recalculating the weight coefficients of the six selected indicators. Using the membership values and weight coefficients, SQI were calculated for the four vegetation restoration measures and the CK. The results indicated an SQI ranking of GU > LC > HS > AO > CK, demonstrating that the GU restoration strategy had the most prominent positive impact on soil quality (**Figure 5**).

**Figure 6:**
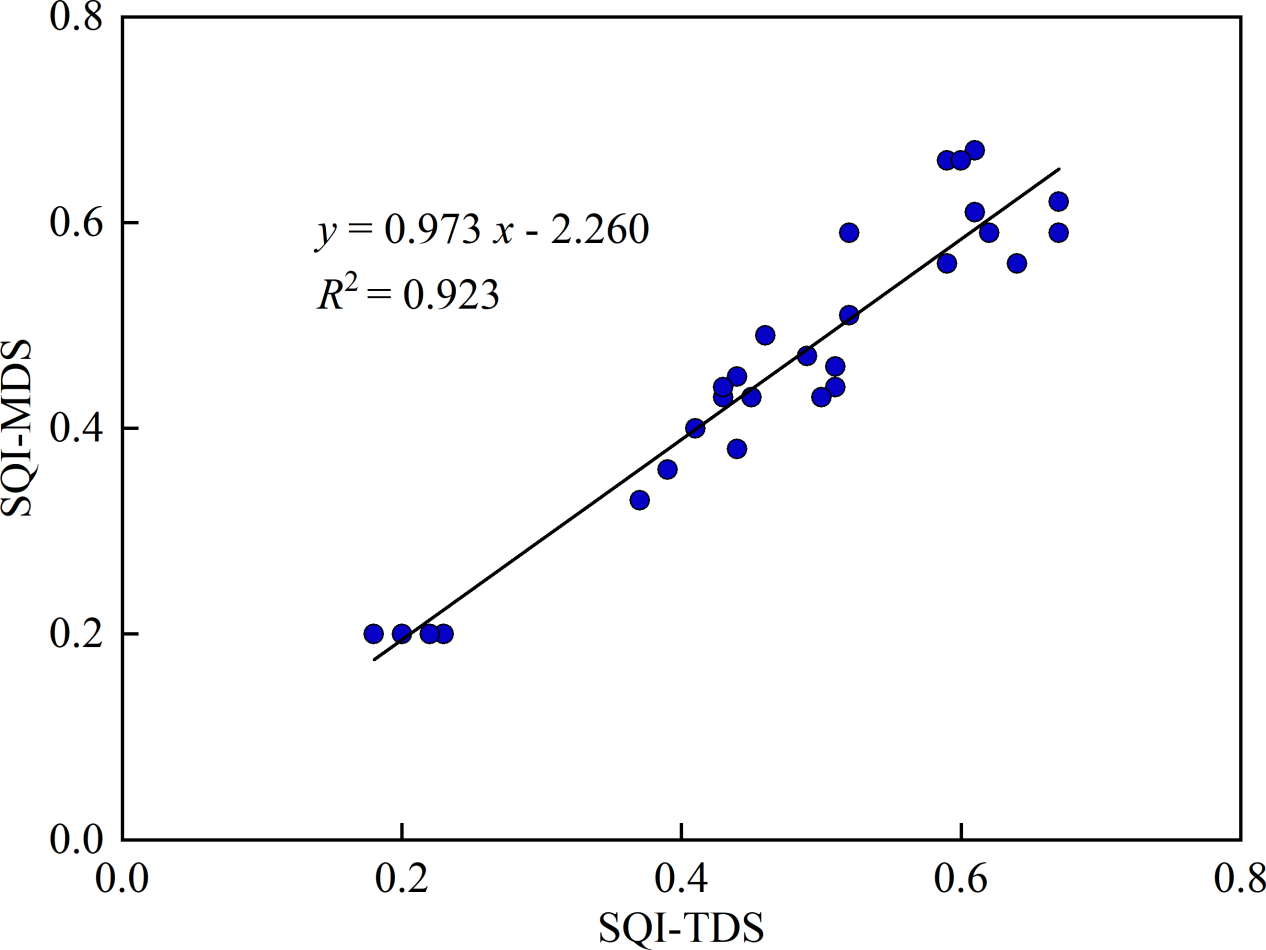
Linear relationship between SQI of the total data set (TDS) and the minimum data set (MDS).

Validating the reliability of the MDS is an important part in soil quality assessment. A regression analysis was performed to compare the SQI derived from the total dataset (SQI-TDS) and the minimum dataset (SQI-MDS), assessing the suitability of the MDS for evaluating soil quality. As shown by the regression results (**Figure 6**), SQI-TDS and SQI-MDS were highly correlated, with a coefficient of determination (R²) of 0.923. This strong correlation confirms that the MDS can effectively replace the TDS, providing a reliable and efficient approach for assessing soil quality under different vegetation restoration strategies in desert PV power stations.

## 4 Discussion

### 4.1 Regulation mechanisms of soil physical properties and moisture dynamics

**Figure 7:**
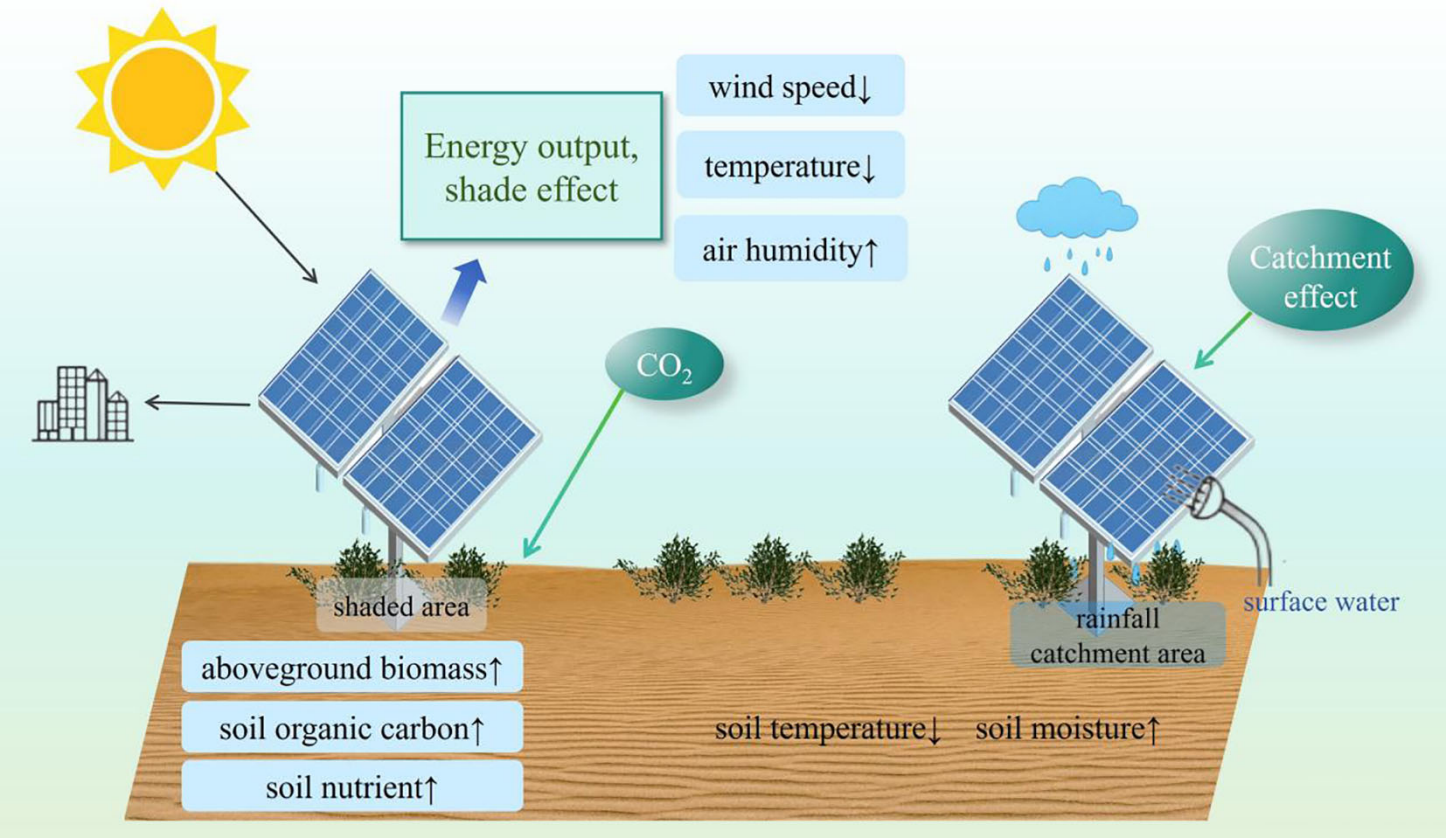
Diagram of the soil-plant feedback mechanism in desert PV power station.

The large-scale installation of PV arrays significantly alters the local light, heat, and water cycles, resulting in varying degrees of microclimatic changes within the PV station (Ravikumar et al., 2017; Yue et al., 2021b; Li et al., 2024; Tan et al., 2025). This study systematically analyzed the effects of different vegetation restoration measures on soil physical properties and moisture dynamics during the operational phase of the PV station, providing key data to enhance our understanding of the interactions between vegetation restoration and the PV station’s microenvironment (**Figure 7**). Our findings indicate that all vegetation restoration measures led to an increase in silt sand content compared to the CK, with increments of 0.2%–2.34% between panels and 0.09%–1.75% under panels (**Table 2**). This aligns with the observations of Yang and Wang (2022), who reported that the secondary airflow induced by PV arrays promotes the deposition of fine particles. The inclined structure of PV panels accelerates wind speed, enhancing the capacity of airflow to transport sand particles, ultimately leading to the accumulation of fine sediments in both the between and under panel areas. Similarly, Tang et al. (2021a) confirmed that wind speed distribution within PV stations significantly influences wind-blown sand movement patterns, which could even pose challenges to the long-term stability of the station. The accumulation of fine particles contributes to improved SP and structural stability, thereby reducing the risk of soil erosion. Notably, the altered soil texture reflects a mixed deposition process driven by both vegetation restoration practices and PV infrastructure operations, because soil texture changed slow with time itself. Additionally, clay particles, due to its high specific surface area and electrostatic properties, plays an important role in soil water retention by adsorbing organic matter and humic substances to form stable organic-mineral complexes. BD and SP are key indicators of soil permeability, aeration, and water-holding capacity (Cherubin et al., 2016). In this study, all vegetation restoration measures reduced BD, with GU exhibiting the lowest BD in under panels (1.47 g·cm⁻³), significantly lower than that of CK (*P < 0.05*). Correspondingly (**Table 2**). Vegetation cover plays an essential role in regulating soil moisture content, as demonstrated by numerous studies (Cherubin et al., 2016; Wang et al., 2018; Shen et al., 2020). Our results indicate that LC, AO, and HS significantly increased MC compared to CK, whereas GU exhibited slightly lower water retention capacity than the other vegetation measures. Furthermore, the shading effect of PV panels altered soil moisture dynamics, leading to a unique regulatory mechanism that deviates from the traditional “vegetation-water trade-off” theory. Zhai et al. (2022) demonstrated that on rainfall redistribution in PV stations demonstrated that the construction of PV stations can significantly enhance soil water storage, mainly attributed to the front gable of PV panels, where runoff from the panel surface is concentrated. Our study confirms that the combined effects of the “rain shadow effect” and “evapotranspiration suppression effect” contribute to enhanced soil moisture retention, with the MC under PV panels being 2.12%–62.15% higher than between panels. Ks and CWC are essential parameters that reflect soil water transmission and retention capabilities (Gootman et al., 2020). Both LC and GU exhibited remarkably higher values for these parameters than other vegetation types and CK, especially under PV panels, where the Ks for LC reached 15.49%, significantly exceeding that of CK (13.39%). These findings suggest that soil moisture conditions are more favorable under PV panels, creating an improved environment for plant growth.

### 4.2 Soil nutrient accumulation and plant-soil feedback mechanism

Vegetation restoration is central to ecological rehabilitation, with soil quality determines the sustainable growth of vegetation. Soil not only directly influences plant growth, but also regulates vegetation community structure and ecosystem functions through nutrient cycling processes. Restoration measures impact soil nutrient accumulation via root secretions, litter deposition and inter-root microbial activities, while the soil nutrient status in turn regulates the vegetation growth and succession process. This plant-soil feedback mechanism is crucial for the ecological restoration and long-term stability of desert PV power stations. Our study revealed that all vegetation restoration significantly increased soil nutrient content. Among these, the LC treatment exhibited the highest TN, TP, and AK compared to CK, increasing by 202.77%, 36.29%, and 430.40%, respectively. Meanwhile, GU demonstrated the highest TK content, reaching 55.16 mg/kg (between panels) and 60.45 mg/kg (under panels), indicating its superior ability to enhance soil fertility (**Figure 3**). This may closely related to the long-term accumulation of litter and its decomposition rate. With the continuous input of litter, the organic matter (e.g., cellulose, lignin, and soluble sugars) was degraded by soil microorganisms and became an important source of energy and nutrients for their growth and metabolism. Although decomposition rates may be low under very dry conditions due to the limitation of moisture limitation on microbial activity, this was not the case in our study. In addition, SOC as the key indicator of soil fertility increased significantly with all vegetation restoration measures. Of these, LC and GU showed the greatest increase in SOC, which was 146.23% and 241.51% higher than that of CK, respectively. This trend indicates that specific plant species regulate SOC dynamics through litter input and decomposition rates. Interestingly, AO exhibited relatively weaker nutrient accumulation capacity, which may be related to its faster growth cycle and higher rate of litter decomposition, thus affecting the SOC stability. Furthermore, changes in the C/N ratio provide key insights into nitrogen cycling processes. The GU exhibited the highest C/N ratio (41.24) indicating potential nitrogen limitation, while LC had a comparatively lower C/N ratio (16.43) showing a higher nitrogen turnover rate. Since the C/N ratio directly influences microbial decomposition rates and plant nitrogen use efficiency, future vegetation restoration strategies in desert PV power stations should prioritize deep-rooted, high-biomass species to maximize soil improvement benefits.

### 4.3 Optimization of soil quality assessment: Application of MDS and strategic recommendations

To enhance the efficiency of soil quality assessment, this study employed PCA to identify six core soil indicators and construct MDS. The results demonstrated that the MDS could effectively replace the TDS, enabling a more efficient and cost-effective approach to soil quality monitoring, particularly suited for ecological management in desert PV power stations (**Figure 6**). Based on the SQI assessment, GU and LC showed the best performance in improving soil physical structure, enhancing water retention, and promoting nutrient accumulation, especially in areas under panels. Although this study primarily focused on soil quality improvements under different vegetation restoration measures, the findings have broader implications for achieving a synergy between renewable energy expansion and ecological conservation. Therefore, for future vegetation restoration efforts in desert PV regions, the following optimized strategies are recommended: 1. Prioritizing large-scale cultivation of GU and LC to improve soil quality and vegetation restoration in PV power station. In the specific implementation process, precise layout can be carried out according to PV microenvironmental conditions, such as planting GU under panels (high infiltration area) and configuring LC between panels (water retention area), to maximize water utilization and soil improvement benefits. 2. Moderate apply biochar and slow-release fertilizers to further optimize soil structure, enhance nutrient use efficiency, and strengthen the long-term supply capacity of carbon, nitrogen and phosphorus. 3. Implement an IoT-based “PV–Soil” integrated monitoring network by combining multispectral remote sensing (to monitor vegetation indices) with soil sensors (to provide real-time data on soil moisture and nutrients). These strategies provide a scientifically grounded and practically feasible pathway for optimizing soil management in desert PV installations, ensuring both ecological and energy sustainability.

## 5 Conclusion

This study aimed to explore effective vegetation restoration strategies for desert PV stations to promote ecological recovery. The results demonstrated that all vegetation restoration measures significantly reduced BD while increasing MC. Among which, LC and GU exhibited superior performance in enhancing soil structure and hydrological function. The shading effect of PV panels created distinct microenvironmental niches, with MC increasing by 19.8-34.6% under panels compared to between panels. In terms of soil nutrients, LC resulted in the highest levels of TN, TP, and AK, highlighting its strong potential for soil improvement. GU, on the other hand, showed the highest TK content and C/N ratio, indicating its important role in soil nutrient cycling. The SQI further confirmed the effectiveness of GU in improving soil conditions, followed by LC, HS, and AO. Overall, vegetation restoration proved to be an effective strategy for improving soil quality in desert PV ecosystems. GU and LC emerged as the most promising species for enhancing soil structure, moisture retention, and nutrient accumulation. Given the ongoing challenges of ecological restoration in desert regions, future research should incorporate multi-omics approaches to unravel the complex plant-soil-microbe interactions. Such insights will be critical for guiding the sustainable management of integrated PV-desert landscapes.

## Data Availability

The original contributions presented in the study are included in the article/supplementary material. Further inquiries can be directed to the corresponding author/s.
